# Evaluation of a new Rapid Antimicrobial Susceptibility system for Gram-negative and Gram-positive bloodstream infections: speed and accuracy of Alfred 60AST

**DOI:** 10.1186/s12866-019-1654-9

**Published:** 2019-11-29

**Authors:** Vanesa Anton-Vazquez, Samuel Adjepong, Cristina Suarez, Timothy Planche

**Affiliations:** 1 Institute of Infection and Immunity. St. George’s University of London, Cranmer Terrace, London, SW17 0RE UK; 2 Department of Medical Microbiology, Southwest London Pathology, St. George’s Hospital Blackshaw Road, London, SW17 0QT UK; 3 Infection Care Group, St George’s University Hospitals NHS Foundation Trust, Blackshaw Road, London, SW17 0QT UK

**Keywords:** Rapid diagnostics. Bloodstream infection. Bacteraemia. Antimicrobial susceptibility testing. Gram-negative bacteria. Gram-positive bacteria

## Abstract

**Background:**

Blood stream infections (BSIs) are a major cause of morbidity and mortality. The time from taking blood cultures to obtain results of antibiotic sensitivity can be up to five days which impacts patient care. The Alfred 60 AST™ can reduce laboratory time from positive culture bottle to susceptibility results from 16 to 25 h to 5–6 h, transforming patient care. To evaluate the diagnostic accuracy of a rapid antimicrobial susceptibility system, the Alfred 60 AST™, in clinical isolates from patients with BSIs and confirm time to results. 301 Gram-negative and 86 Gram-positive isolates were analysed directly from positive blood culture bottles following Gram staining. Antimicrobial susceptibility results and time-to-results obtained by rapid Alfred 60 AST system and BD Phoenix were compared .

**Results:**

A total of 2196 antimicrobial susceptibility test results (AST) were performed: 1863 Gram-negative and 333 Gram-positive. AST categorical agreement (CA) for Alfred 60 AST™ was 95% (1772/1863) for Gram-negative and 89% (295/333) for Gram-positive isolates. Gram-negative CA: ampicillin 96% (290/301); ciprofloxacin 95% (283/297); ceftriaxone 96% (75/78); meropenem 97% (288/297); piperacillin-tazobactam 95% (280/295); gentamicin 94% (279/297) and amikacin 93% (277/298). The median time to susceptibility results from blood culture flagging positive was 6.3 h vs 20 h (*p < 0.01*) for Alfred system vs BD Phoenix™.

**Conclusion:**

Alfred 60 AST system greatly reduced time to antimicrobial susceptibility results in Gram-negative and Gram-positive BSIs with good performance and cost, particularly for Gram-negative bacteraemia.

## Background

Blood stream infections (BSIs) are worldwide, a major cause of morbidity and mortality [1]. The clinical laboratory plays a key role in the diagnosis of BSIs and provides antimicrobial susceptibility results which are crucial in clinical decision-making. Blood cultures remain the main way to identify blood stream infection. Despite optimisation in laboratory workflows that have decreased time-to-result (Anderson, [2]), the time from taking a blood culture to obtaining a result of antimicrobial susceptibilities can be up to 5 days [3]. The time to positivity, time from blood culture collection to bottle flagging positive, is typically 12 to 24 h [1]**.** The subsequent mean time to identify bacterial species and obtain antimicrobial susceptibilities, using standard methods, is a further 36 h [4] .

Phenotypic antimicrobial susceptibility tests (AST) generally relies on detecting bacterial growth in the presence of antibiotic. Standard methods of testing used in routine diagnostic laboratories for patient care include broth or agar micro-dilution, disc diffusion or antibiotic gradient strips, which require around 24–72 h to complete [5]. A number of automated systems for testing antimicrobial susceptibility are increasingly used such as BD Phoenix™ (Becton Dickinson, USA), VITEK-2™ (BioMerieux, France) and MicroScan™ (Beckman, USA) which have a time to results ranging between 12 and 24 h after positive culture [6]**.**

The time to clinically useful antimicrobial susceptibility data has an impact on patient care either to change to an effective antibiotic in the case of a resistant isolates or to focus to a narrower spectrum antibiotic for susceptible isolate. The development of rapid diagnostics is a key aim in the control of the rise in antibiotic resistance [7]. The use of genotypic methods such as nucleic acid amplification tests to detect antibiotic resistance in routine blood cultures has not been widely adopted due to high cost [8], lack of sensitivity when using blood direct from the patient [9] and inability to detect many resistance mechanisms and hence “rule-in” the use of antibiotics when no resistance marker is detected [10]. This highlights the need to increase the speed of phenotypic methods to shorten the time to obtain antimicrobial susceptibility results [11].

The Alfed 60 AST™ (Alifax, Italy) is a CE marked automated commercial laser-scattering based in-vitro diagnostic (IVD) providing antimicrobial susceptibility results directly from positive blood culture bottles within 4–6 h. The Alfred 60 AST™ uses light scatter to detect bacterial growth in a liquid culture broth. In brief, 30 μL of blood from a positive blood culture is inoculated into a broth that is incubated, then light scatter is measured until it reaches a turbidity of 0.4–0.6 McFarland. A series of tubes containing antibiotics are then robotically inoculated and bacterial growth measured by light scatter providing real time bacterial growth curves. Growth curves of bacteria over 3–5 h in the tested antibiotics are compared to growth a positive control (no antibiotic) and percentage of inhibition of growth (%PIC) is calculated. The %PIC is compared between control and antibiotic tubes and reported as resistant, intermediate and sensitive categories, according to the range of inhibition (Sensitive result: Inhibition of growth > 65%; Intermediate: Inhibition of growth between 65 and 50%; Resistant: Inhibition of growth < 50%). To grant a reliable detection of the growth inhibition (keeping as reference the “Reference Vial”) a proper growth must be detected (≥ 700.00 CFU/ml), if not the test is not considered valid.

**Fig. 1:**
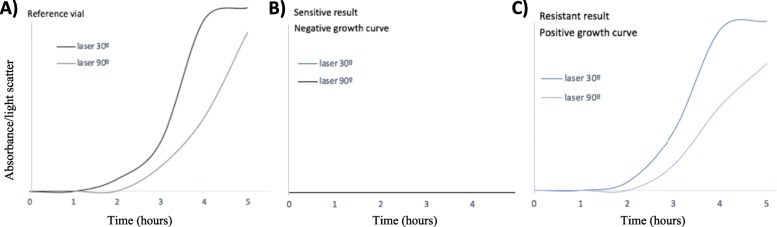
A) represents the standard growth curve without presence of antibiotic (curves are displayed representing the light scattered signals received by photodetectors placed at 30° and 90° from the laser beam). B) represents a sensitive or susceptible antimicrobial results. C) Represents a resistant result, in the presence of antibiotic the growth curve is positive, comparable to the reference vial

The system is based on a light scattering technique, A light laser beam which is oriented trough a sample and two photo detectors placed at 30° and 90° receive signals generated by the light scattered by the bacteria present in the vial. Growth kinetic curves are obtained for each detector and displayed in two curves. Double reading channel (2 detectors) is important for samples with high initial turbidity level. If 1 detector (the 1st one) becomes saturated due to the high sample’s turbidity then the 2nd detector is able to detect signals. Finally, a mathematical algorithm selects the best reading detector channel for the calculation of the inhibition of growth. (Fig. 1)**.**

The panel of antibiotics tested can be predefined by users. Hands on time for each run is about 15 min. In addition, the system requires a daily set up of approximately 10 min and a weekly maintenance wash out of 30 min. Up to 60 individual tests can be performed in a run, the number of isolates tested depends on the number of antibiotics tested (for example 10 isolates may be tested for 6 antibiotics each).

The time to result is unaffected by the number of antibiotics or samples tested. Alfred 60 AST recognizes each reference vial followed by its respective antibiotic vials as a single assay and has the capacity to initiate multiple single susceptibility assays at the same time for different isolates.

The purpose of this study was to compare the accuracy and speed of results of the Alfred 60 AST™ with the BD Phoenix™ (Becton Dickinson, USA) automated susceptibility testing system, a broth-based microdilution test that utilizes a redox indicator to enhance the detection of organism growth, which is the standard of care in our routine diagnostic laboratory.

## Results

### Antimicrobial susceptibility results of Alfred 60 system

Antimicrobial susceptibility results were performed for 387 clinical positive blood culture bottles that were monomicrobial on Gram stain (301 Gram-negative and 86 Gram-positive) with a total of 2196 individual antimicrobial test results (1863 Gram-negative and 333 Gram-positive). Eighteen samples (5%) were excluded from the analysis. Excluded samples comprised 3 polymicrobial samples that were not detected during Gram stain and 15 non valid results included 10 system technical failure before AST performance and 5 failures of no bacterial growth in the reference vial on Alfred 60 AST system, including: 1 *Acinetobacter baumanni*; 1 *Achromobacter spp*; 1 *Bacteroides fragilis*; 1 *Pseudomonas aeruginosa*; 1 *Klebsiella oxytoca*.

71% (213/301) of the Gram-negative and 43% (37/86) of the Gram-positive isolates were resistant to at least one antibiotic. 9% (28/301) of the Gram-negative organisms were identified as Extended-spectrum beta-lactamases (ESBLs) as defined by BD Phoenix™ expert rules for resistance. Fig. 3**.**

There were a total of 301 valid Alfred 60 AST results for Gram-negative monomicrobial isolates. Data were available on the following organisms: 209 *E.coli*; 57 *Klebsiella spp*; 10 *P. aeruginosa*; 12 *Proteus spp*; 7 *Enterobacter spp*; 6 *Morganella morgani*. A total of 1863 AST results were produced and compared with BD Phoenix™**.**

### Alfred 60 AST agreement for gram-negative bacteria

Overall categorical agreement for Alfred 60 AST was 95% (1772/1863) for Gram-negative isolates (55 major errors, 31 very major errors and 5 minor errors). The highest agreement was observed with meropenem 97% (288/297); ampicillin 96% (290/301); ciprofloxacin 95% (283/297); ceftriaxone 96% (75/78) and piperacillin-tazobactam 95% (280/295) Lower agreement was seen for amikacin 93% (277/298) and gentamicin 94% (279/297). Out of a total of 55 major errors, 65% (36/55) involved *E.coli* against amikacin 13; gentamicin 8; piperacillin-tazobactam 6; meropenem 3; ampicillin 4; ciprofloxacin 2 and 20% (11/55) involved *Klebsiella spp*. against ciprofloxacin 3; meropenem 3; amikacin 2; piperacillin/tazobactam 2. Very major errors were seen with ciprofloxacin 6; ampicillin 7; gentamicin 7; piperacillin/tazobactam 5; meropenem 3; ceftriaxone 2 and amikacin 1. Tables 1 and 2.

**Table 1:** AST results of the Alfred 60AST™ system compared to BD Phoenix™ system for each antibiotic. Column 2 shows the total number of susceptibility tests done with both methods; columns 3 to 6 the total number of tests for which there was agreement within each category and in total; columns 8 to 10 the number of discrepancies; S = susceptible = intermediate, R = resistant, CA = Categorical agreement, 95% CI = 95% confidence interval

		No. of category agreements					No. of discrepancies		
Antimicrobial agent	No. of AST results	S	R	I	CA Total	CA % (95% CI)	Minor	Major	Very major
Gram-negative antimicrobials									
Ampicillin	301	88	202	0	290	96 (94–98)	0	4	7
Amikacin	298	273	2	2	277	93 (89–96)	3	17	1
Ciprofloxacin	297	222	61	0	283	95 (92–97)	1	7	6
Ceftriaxone	78	64	11	0	75	96 (89–99)	0	1	2
Gentamicin	297	240	38	1	279	94 (91–96)	1	10	7
Piperacillin/Tazobactam	295	263	16	1	280	95 (92–97)	0	10	5
Meropenem	297	284	4	0	288	97 (94–99)	0	6	3
Overall agreement	1863	1434	334	4	1772	95 (94–96)	5 (0.3%)	55 (3%)	31 (2%)
Gram-positive antimicrobials									
Cefoxitin	75	34	35	0	69	92 (83–97)	0	4	2
Cindamycin	75	41	17	2	60	80 (69–88)	1	9	5
Teicoplanin	86	79	0	1	79	92 (84–97)	1	6	0
Vancomycin	86	76	1	0	77	90 (81–95)	0	9	0
Ampicillin	11	9	1	0	10	90 (59–100)	0	1	0
Overall agreement	333	239	54	3	295	89 (85–92)	2 (0.6%)	29 (9%)	7 (2%)

**Table 2:** AST results of the Alfred 60 AST™ system compared to BD Phoenix™ system for 301 Gram-negative and 86 Gram-positive organisms included in the study. Column 2 refers to the total number of susceptibility tests done with both methods; colum 3 refers to the number of tests for which there was agreement between both tests. S = sensitivie, I = intermediate, R = resistant, CA = Categorical agreement, 95% CI = 95% confidence interval. CoNS – Coagulase negative staphylococcus, 95%CI- 95% confidence interval

					No. of discrepancies		
	No. of Isolates	No. of AST results	No. of CA	CA % (95% CI)	Very major	Major	Minor
Gram-negative organisms							
E.coli	209	1291	1242	96 (95–97)	9	36	4
Klebsiella spp	57	351	330	94 (91–96)	10	11	1
P.mirabillis	12	79	69	87 (78–84)	6	3	0
P.aeruginosa	10	61	58	95 (86–91)	0	3	0
E.cloacae	7	43	37	86 (72–95)	0	6	0
M.morgani	6	38	36	95 (82–99)	0	2	0
Overall Gram-negative	301	1863	1772	95 (94–96)	31	55	5
Gram-positive organisms							
S.epidermidis	26	104	98	94 (88–98)	3	2	1
S.aureus	21	84	66	79 (68–87)	1	17	0
S.hominis	15	60	54	90 (79–96)	2	4	0
CoNS other	13	52	48	92 (81–97)	1	3	0
E.faecalis	10	30	27	93 (73–98)	0	3	0
E.faecium	1	3	2	67 (10–100)	0	0	1
Overall Gram-positive	86	333	295	89 (85–92)	7	29	2

### Alfred 60 AST agreement for gram-positive bacteria

A total of 86 valid Gram-positive organisms were analysed giving a total of 333 Alfred 60 AST results. The overall categorical agreement for Alfred 60 AST was 89% (295/333) for Gram-positive samples. The highest agreement was observed with cefoxitin 92% (69/75) and teicoplanin 92% (79/86) whilst clindamycin and vancomycin showed the lowest one 80% (60/75) and 90% (77/86) respectively. There were 29 major errors, 7 very major errors and 2 minor errors. Seventeen of the major errors (68%) involved *S. aureus* tested clindamycin 6, teicoplanin 6 and vancomycin 5. The remaining 12 major errors involved *CoNS* and *Enterococci Spp* against vancomycin and cefoxitin. Five of the very major errors involved *CoNS* tested against clindamycin and two involved *S. aureus* and *Staphylococcus epidermidis* tested against cefoxitin. Tables 1 and 2.

### Time to results

The median time to susceptibility results from flagging positive blood cultures was 6.3 h (IQR = 5.25–8.4) for Alfred 60 AST system compared to the 20 h (IQR = 16–24) for BD Phoenix™. As a result, a reduction in the turnaround time by 13 h when compared to BD Phoenix™ was observed.

In terms of “hands-on-time”, in our experience, an average time of 20 min were required to load the samples on the Alfred rapid system, comparable to 15 min for BD Phoenix™ system. Table 3**.**

**Table 3:** Turnaround time for results from blood culture positivity: Median Time (in hours) and Interquartile range from positive blood culture to results of Alfred vs BD Phoenix™. Time in hours from positive blood culture to susceptibility results

	Alfred 60 AST	BD Phoenix™ system	Alfred™ vs BD Phoenix™
Time to results from positive blood culture (h)	6.3 h (IQR, 5.25–8.25)	20 h (IQR, 16–24)	p < 0.01
Technician “hands-on” time (min)	20 min (IQR, 10–30)	15 min (IQR, 10–20)	
Technique Characteristics	Direct culture bottle	Agar plate subculture (4 h)	

## Discussion

This study shows the performance of Alfred 60 AST™ system, which significantly reduced the time to antimicrobial susceptibility results. The overall categorical agreement for Alfred 60 AST system was 96% for Gram-negative and 90% for Gram-positive organisms. These results are broadly comparable to an earlier smaller study reported by Barnini et al [12], which compared the Alfred 60 AST™ with VITEK-2™, and reported a categorical agreement of 91% for enterobacteraciae and 94% for staphylococci. Although similar, differences in the performances may be due to the choice of antibiotics tested, Barnini et al tested ceftazidime and gentamicin, but did not test amoxicillin and ciprofloxacin.

Compared to the BD Phoenix, the overall categorical agreement for Alfred 60 AST system was 96% for Gram-negative and 90% for Gram-positive organisms. Previous studies report the accuracy of the BD Phoenix™ system for susceptibility results compared against agar dilution and manual disc diffusion, showing categorical agreement of 97.5% [13] and 95% (Donay, 2004) respectively.

Alfred 60AST™ system achieved a performance of over 95% CA compared to the BD Phoenix™ for all antibiotics except amikacin and gentamicin. However, this new system showed slightly lower performance for Gram-positive isolates and for glycopeptides and clindamycin.

In our study, the average hands-on time per specimen was not different from routine testing, however the time to deliver susceptibility results has been significantly reduced compared to routine standard automated system (BD Phoenix™). The times to AST results were similar to the times noted in a previous study using the Alfred 60 AST™ system and other the Accelerate Pheno™ system (Accelerate Diagnostics, USA) [12] [15] [16] [17]. Given high categorical agreement between the BD Phoenix™ and Alfred 60 AST™ and the 16 h reduction in time to results there are compelling clinical benefits of the Alfred AST 60™.

The Accelerate Pheno™ system is an alternative rapid phenotypic testing system. In studies using similar AST comparator techniques, an agreement of 93.3% [18], 94.9% [19] and 96.4% respectively [15] has been reported. Showing very similar diagnostic performance to the Alfred AST 60™. The median time to AST results from positive culture bottle to AST result for the Accelerate Pheno™ system is reported as 10.7 [8.6–12.8] hours [15]. It should be noted that an advantage of the Accelerate Pheno™ system is that it identifies the species of the isolate in addition to the sensitivity pattern. For this reason, we needed to perform a rapid identification with a MALDI-TOF MS at the same time as the Alfred 60 AST™.

A specific advantage of the Alfred 60 AST™ system versus other rapid phenotypic susceptibility methods is the capacity to accommodate between 7 and 9 isolates per run, the number of samples may be varied according to the customized antibiotic panel chosen by the user and a satellite module provides extra capacity to handle up to a total of 20 isolates per day. Furthermore, new samples can be loaded during an ongoing run without interfering previous loaded isolates.

The cost of performing rapid phenotypic sensitivity testing for some systems has been regarded as high and seen as a barrier to introducing these tests. We estimate the additional reagent costs for the Alfred 60 AST™ of €2–4 per antibiotic tested [20] or €30–40 per isolate, with relatively minimal laboratory hands on time. This cost is considerably lower than other rapid testing systems and we believe could be justified by the clinical benefits.

One of the limitations of the system is it will not perform antimicrobial susceptibility testing on polymicrobial samples, although Gram-staining before using the Alfred 60 AST™ meant polymicrobial samples were relatively uncommon in these samples. Another possible drawback is the absence of MIC values, which are not provided on the susceptibility report, that may occasionally be relevant for specific therapeutic decisions [21], however most clinical decisions can be made simply with an S/I/R result. Another limitation lies on the inability of performing susceptibility testing of *Acinetobacter baumannii* or *Stenotrophomona maltophila* which are emerging as a challenging to treat nosocomial pathogens [22]. Furthermore, there are inconsistencies with the concentration of cefoxitin in the Alfred 60 AST™ panel. The EUCAST breakpoint for cefoxitin is 4 mg/L for *S. aureus* and *S. lugdunensis* and 8 μg/L for *Staphylococcus saprophyticus*, whilst Alfred 60AST™ breakpoint for all coagulase negative Staphylococci is 8 μg/L.

Limitations of the study were that although broth microdilution is considered the “gold standard” technique for antimicrobial susceptibility evaluation, this is rarely routinely available in practice in clinical laboratories. The BD Phoenix™ system is a well validated automated AST system [13]. Similar methodologies of comparing rapid diagnostic tests with automated AST systems in place of broth or agar dilution has been used in majority of published studies [19] [16] [15] [17] [18] [13].

We note the relatively small proportion of multi-resistant microorganisms in our particular setting and the fact that more than half of the susceptibility testing for Gram-negative isolates were performed for *E.coli* blood stream infections. Furthermore, a smaller number of Gram-positive organisms were tested compared to the Gram-negative organisms.

Even though there is clinical utility in rapid antimicrobial sensitivity results we would still recommend isolates have further extensive testing with another system, such as the BD Phoenix™ or a VITEK™ 2, to give a complete sensitivity pattern to a wider range of antibiotics and give results for minimum inhibitory concentrations. Since, BD phoenix shows more accuracy specially for gram positive bacteria.

The implementation of the Alfred 60 AST™ system in the laboratory routine workflow, will provide rapid susceptibility results, that effectively communicated to clinicians might result in a prompt change to appropriate antimicrobial treatment, antimicrobial resistance reduction and improved infection control [23] [24] [25].

## Conclusions

In conclusion, Alfred 60 AST™ system has demonstrated an improvement in the turn-around time of antimicrobial phenotypic susceptibility results in Gram-negative and Gram-positive blood stream infections in comparison with automated AST techniques and conventional AST testing.

Whilst the system provided a notably good performance and high accuracy for Gram-negative bacteria, it still needs improvements in Gram-positive AST results and is less dependable for yeast detection [20].

Prospective studies are needed to further analyse the impact and effects of the rapid diagnostic techniques among bacteraemia patients and consequences of the introduction of new available rapid automated systems on antibiotic prescription against BSI and on clinical patient pathways.

## Methods

### Study setting

A prospective evaluation was conducted directly from blood cultures, between January and May 2018 in the microbiology laboratory of South West London Pathology in London. The diagnostic laboratory processes approximately 3500 positive blood cultures per year. All blood culture samples were initially incubated on the BD BacTec FX400 (Becton Dickinson, USA). Blood culture bottles, including adult and paediatric samples, that flagged positive were then tested in parallel for antimicrobial susceptibility by Alfred 60 AST and by routine testing on the BD Phoenix™, after Gram staining performance to determine a Gram-positive or Gram-negative antibiotic panel required and exclude the polymicrobial samples.

**Fig. 2:**
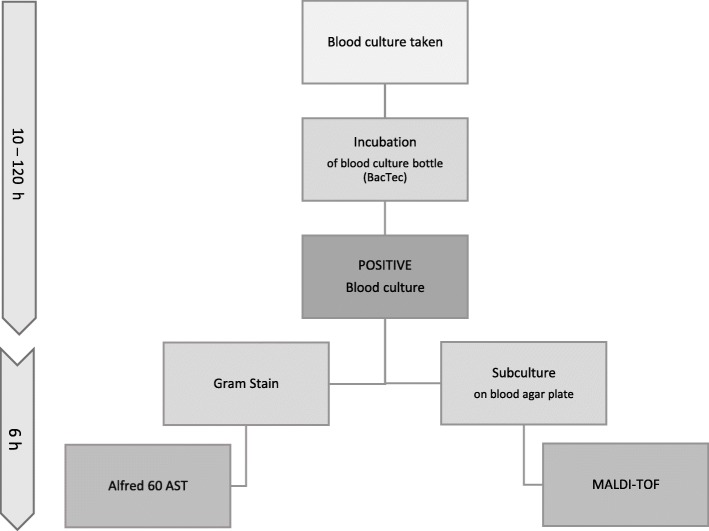
Flowchart showing laboratory workflow followed along the rapid susceptibility system (Alfred 60 AST) evaluation. Once a blood culture bottle flagged positive following incubation, Gram-stain was performed in order to distinguish between Gram-positive and Gram-negative microorganism and exclude the polymicrobial ones, which will allow to select the appropriate antibiotic panel and a direct blood sample from the bottle was loaded onto the rapid system. In parallel, the identification results were obtained by MALDI-TOF, performed from isolated colonies obtained by 4 h agar plate subculture. The mean total time from positive blood culture to identification and susceptibility results was 6 h (approx)

Bacterial species were identified by Bruker Biotyper matrix assisted laser desorption ionization-time of flight mass spectrometry (MALDI-TOF MS, Germany), this technique was performed, as per routine laboratory practice, from isolated colonies obtained following 4 h agar plate subculture, in order to fully interpret the results of the Alfred 60AST with species ID**.** This meant the species of the samples were identified before the Alfred 60AST result was available. (Fig. 2).

**Fig. 3:**
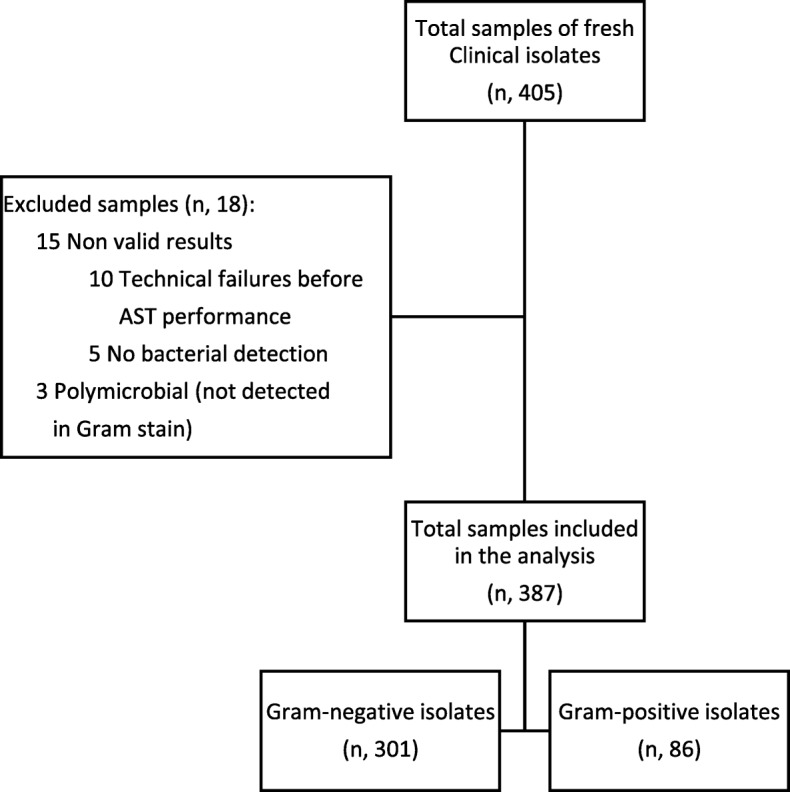
Flowchart of study population. A total of 387 (301 Gram-negative and 86 Gram-positive) valid monomicrobial clinical isolates after 18 excluded samples. Excluded samples comprised 3 polymicrobial and 15 non valid results included 10 system technical failure and 5 no bacterial detection and subsequently no AST performance

### Alfred 60 AST system

The Alfred 60 ST was performed according to the manufacturer’s instructions on blood culture bottle that had flagged positive on the BD BacTec FX system, this including running weekly controls with *Pseudomonas aeruginosa* 27,853; *E.coli* 25,922; *E.coli* 35,218; *S.aureus* 29,213; *E.faecalis* 29,212; *E. faecalis* 51,299. 30 μL of the sample was inoculated into an enrichment broth and loaded onto the instrument. Each vial of antibiotic contains 45 mg of a preparation of lyophilized antibiotic that required to be dissolved in 2 mL of regenerating solution, as per manufacturer’s specifications. Some antibiotics, including amikacin, piperacillin-tazobactam and meropenem, required different specific antibiotic vials for testing *Enterobacteriaceae* and *Pseudomonas Spp.* As the identity of organism is unknown when the Alfred 60 AST system was set up, a combined panel of antibiotics with both concentrations was used. When the identity of the organism was known the appropriate antibiotic result was reported.

### Antimicrobial agents

The following antibiotic panel was performed on Alfred 60AST™ for all Gram-negative organisms (*Enterobacteriaceae* and *Pseudomonas spp*).: ampicillin, amikacin, ciprofloxacin, gentamicin, meropenem, and piperacillin-tazobactam. Ceftriaxone was included on the *Enterobacteriaceae* panel later in March 2018 and tested in 78 isolates. For Gram-positive organisms the antibiotic panel included: ampicillin, cefoxitin, clindamycin, teicoplanin and vancomycin. Purity plates were set up both on CLED agar and blood agar plates and examined the next day to confirm that the inoculum was not mixed.

### Comparator AST technique

The BD Phoenix™ automated susceptibility testing system CE marked IVD (BD Diagnostics, Sparks, MD, USA) (software version 5.02H/4.11B) was used as the routine laboratory method according to the manufacturer instructions, including running weekly controls with *Pseudomonas aeruginosa* 27,853; *E.coli* 25,922; *E.coli* 35,218; *S.aureus* 29,213; *E.faecalis* 29,212; *E. faecalis* 51,299 using cartridge PMIC-96 for Gram-positive and NMIC-417 for Gram-negative isolates.

### Interpretation and comparison of results

Isolates were classified as susceptible, intermediate or resistant (S/I/R) according to interpreted reports provided by Alfred 60AST™ and BD Phoenix™. The final report interpretation of susceptibility results provided by Alfred 60 AST™ are based on bacterial growth curve analysis Fig. 1**.**

Each sample result by Alfred 60 AST system was compared against BD Phoenix™. Categorical agreement (CA) was defined as agreement of test results interpreted within the same susceptibility category(S/I/R).

Discordant results were categorized as very major error (VME, reported susceptible on Alfred AST60™ when reported resistant Phoenix™), major error (ME, reported resistant when susceptible) or minor error (mE, reported intermediate when susceptible or resistant). The criteria for antimicrobial susceptibility testing proposed by Jorgensen has been used [26] [27]**.**

### Time to AST results

The time to AST was defined as the time between the culture bottle flagging positive on the BacTec and the availability of the AST result by Alfred 60 AST system or BD Phoenix™. Wilcoxon test was performed for comparison of median AST time analyses, considering *p* < 0.05 as a significant value.

SPSS (Statistical Package for the Social Sciences, IBM, USA) version 25.0 statistics software was used for all analyses.

## Data Availability

Raw data used in the study are provided in the form of excel spreadsheets on the SGUL data repository. https://sgul.figshare.com/s/c8746f6e57980278bb4f DOI 10.24376/rd.sgul.9782243

## Abbreviations

AST: Antimicrobial susceptibility testing
BSI: Blood stream infections
CA: Categorical agreement
*CoNS*: *Coagulase negative Staphylococci*
ESBLs: Extended-spectrum beta-lactamases
EUCAST: European Committee on Antimicrobial Susceptibility Testing
MALDI-TOF: Matrix-Assisted Laser Desorption/Ionization-Time Of Flight
ME: Major error
mE: minor error
MIC: Minimum inhibitory concentration
S/ I/ R: Susceptible/ Intermediate/ Resistant
UK HRA: United Kingdom Health Research Authority
VME: Very major error
